# Et Ceteras

**Published:** 1847-05

**Authors:** 


*Et Ceteras.*—Prof. Martyn Payne has sailed for Europe. He has also just issued a new work on the Institutes of Medicine, from the press of the Harpers.—*Mala Praxis.* Dr. William Robertson, of Hanover, Ohio, has been mulcted in the sum of $2,250 for maltreatment of a dislocated knee, which made it necessary to amputate. Had the unfortunate patient died we suppose no malpractice would have been alleged.—Priesnitz, the inventor of the "water-cure" is dangerously sick; neither Paracelsus, nor Hahnemann, nor Priesnitz, have yet found the elixir of life—"it is appointed unto all men once to die."—In Boston the public Schools are beginning to introduce chairs with backs; here the boys still carry their own backs.—Dr. Horace Wells, of Hartford, Conn., has published a pamphlet to prove himself the discoverer of the application of nitrous oxide gas ether, and other vapors, to surgical operations. Dr. Jackson and Mr. Morton are also each claiming priority. Will it not be prudent in all these gentlemen to wait until its value is more fully ascertained, or at least its safety; they may yet find themselves quarreling over a marrowless bone.—Green, *vs.* Reese, or "the bronchitis war" still rages. In one of his last shots Reese sends home the charge that in February 1847, Green was excluded from the fellowship of the New-York Medical and Surgical Society, under charge of unprofessional conduct in reference to this very book on Bronchitis, and this by a summary vote of its members.—The Grand Jury at Montreal have presented the Medical Profession as being guilty of admitting persons daily to the practice of Medicine in Canada, who are not qualified.—A new school has been chartered by the legislature of "Massachusetts, called the "Boylston Medical School," and the Corporation are appointed.—Much Diarrhœa is prevailing in the Army at Vera Cruz, but at the last accounts no vomito.—The Censors elected by the State Medical Society for the Western District, are Drs. William Taylor, Bryant Burwell and John Coats.

